# The Association Between Cholecystectomy and the Risk for Fracture: A Nationwide Population-Based Cohort Study in Korea

**DOI:** 10.3389/fendo.2021.657488

**Published:** 2021-05-27

**Authors:** Eun Ji Lee, Cheol Min Shin, Dong Ho Lee, Kyungdo Han, Sang Hyun Park, Yoo Jin Kim, Hyuk Yoon, Young Soo Park, Nayoung Kim

**Affiliations:** ^1^ Department of Internal Medicine and Seoul National University Bundang Hospital, Seongnam, South Korea; ^2^ Department of Statistics and Actuarial Science, Soongsil University, Seoul, South Korea; ^3^ Department of Medical Statistics, College of Medicine, Catholic University of Korea, Seoul, South Korea

**Keywords:** cholecystectomy, vitamin D deficiency, osteoporosis, fracture, risk

## Abstract

**Objectives:**

To evaluate the risk of fracture in individuals with a history of cholecystectomy in Korean population.

**Methods:**

Individuals (n = 143,667) aged ≥ 40 y who underwent cholecystectomy between 2010 and 2015 and the controls (n = 255,522), matched by age and sex, were identified from the database of the Korean National Health Insurance Services. The adjusted hazard ratio (aHR) and 95% confidence interval (CI) of fracture were estimated following cholecystectomy, and a Cox regression analysis was performed.

**Results:**

The incidence rates of all fractures, vertebral, and hip fractures were 14.689, 6.483 and 1.228 cases per 1000 person-years respectively in the cholecystectomy group, whereas they were 13.862, 5.976, and 1.019 cases per 1000 person-years respectively in the control group. After adjustment for age, sex, income, place of residence, diabetes mellitus, hypertension, dyslipidemia, smoking, alcohol drinking, exercise, and body mass index, patients who underwent cholecystectomy showed an increased risk of all fractures, vertebral fractures, and hip fractures (aHR [95% CI]: 1.095 [1.059-1.132], 1.134 [1.078-1.193], and 1.283 [1.139-1.444] for all fractures, vertebral fractures, and hip fractures, respectively). The risk of vertebral fractures following cholecystectomy was more prominent in the young age group (40 to 49 y) than in the old age group (≥ 65 y) (1.366 [1.082-1.724] *vs.* 1.132 [1.063-1.206], respectively). However, the incidence of hip fractures following cholecystectomy was not affected by age.

**Conclusion:**

Individuals who underwent cholecystectomy have an increased risk of fracture. In the younger population, the risk of vertebral fractures may be further increased following cholecystectomy.

## Introduction

Cholecystectomy is one of the most common surgical procedures and patients who have undergone cholecystectomy can easily be encountered in clinical practice. In Korea, the cholecystectomies per 100,000 persons were 149 cases in 2018, which increased at the rate of 5.9% over 5 years. Cholecystectomy expense was ranked fifth of 33 major types of surgery. The most common cause of cholecystectomy was gallstones, followed by cholecystitis (1).

Laparoscopic cholecystectomy is the gold standard for the treatment of gallstone diseases after its initiation and is generally considered a safe and effective procedure that can be performed with minimal risk (2–7). However, the delayed medical outcomes of cholecystectomy are not fully understood. New emerging evidence suggests that cholecystectomy increases the risk of certain diseases such as metabolic syndrome or non-alcoholic fatty liver disease (NAFLD) (8–12).

Fractures are also common illnesses, which refer to broken bones caused by an external impact and a state in which bone continuity is completely or incompletely broken. Owing to its incidence and increased social burdens in the aging population, fractures are considered a major health problem worldwide (13, 14). The number of patients who received medical treatment for fractures in Korea increased by 250,000 to 1.23 million in 2016 compared to 1.98 million in 2012, and the medical cost increased by 41.3 billion won from 1.12 trillion won in 2012 to 1.55 trillion won in 2016 (15).

As mentioned above, history of cholecystectomy and fractures are both prevalent in the general population; we speculated that fracture might be more prevalent in patients undergoing cholecystectomy. Osteoporosis is a systemic disease characterized by low bone marrow density (BMD) and micro-architectural deterioration of the bone tissues. It is one of the most important causes of fracture, and low bone density *per se* is a risk factor for almost all the types of fracture (13, 14, 16–19). In several previous studies, lower vitamin D level and lower BMD was noted in patients with previous cholecystectomy (20, 21). On the other hand, other studies showed a lower vitamin D level but similar BMD following cholecystectomy (22, 23). The discrepancy may be due to the small sample size of these studies. To date, there is no report that evaluated the relationship between cholecystectomy and fracture, which is the clinical end point of osteoporosis.

Considering this, we investigated the association between cholecystectomy and fractures compared to an age- and sex-matched control group. For the analysis, a national-population-based dataset obtained from the Korean National Health Insurance Service was used.

## Materials and Methods

### Database

This study was designed as a nested case-control study that used data form the Korean National Health Insurance Corporation (NHIC) database, in which approximately 97% of the Korean population is registered. The NHIC is a single public health insurance program managed by the Korean government, and recommends subscribers to receive a comprehensive medical examination at least biennially from the age of 40 y. It contains beneficiary information such as age, sex, place of residence, income, and medical claims information including disease codes and procedure codes. Researchers can approach the NHIC database in a specific time and place, after an approval by the official review committee.

All procedures involving human participants were performed in accordance with the ethical standards of the institutional and national research committees and the 1964 Helsinki declaration including its later amendments or comparable ethical standards. This study was approved by the Institutional Review Board of Seoul National University Bundang Hospital (IRB number: X-2003/601-904). The requirement for informed consent was waived because the study was based on routinely collected medical claim data.

### Study Population

From the total population of the Republic of Korea, 345,940 participants who were older than 40 years and who underwent cholecystectomy between 2010 and 2015 were recruited. Cholecystectomy operations were identified by the corresponding insurance claim codes (Q7380). For the control population, 679,740 participants were selected as a 1:2 age- and sex-matched control for the 339,870 participants who underwent cholecystectomy. For the participants who had undergone cholecystectomy, their index dates were defined as the date of cholecystectomy. For controls, it was assigned to each control as the index date of the matched case. Among the cases and the matched controls (n = 1,019,610), the individuals who had medical examination data within 2 y from the index date were selected (n = 520,445) and excluded the patients <40 y old (n = 65,130), or those with previous history of fracture (n=47,015), or those who developed fractures within 1 y following the enrollment were excluded (n = 9,111). Finally, we analyzed 143,667 cases and 255,552 controls in this study. The flow chart of the study set is presented in **Supplementary Figure S1**.

Medical record data and standardized self-reporting questionnaires were evaluated. Questionnaires at baseline included age (y), sex, smoking, drinking, physical activity and residency. Anthropic variables including blood pressure, height, weight, waist circumference, and values of serum fasting glucose and total cholesterol (mg/dl) were collected from the database.

### Study Outcomes

The primary endpoint was newly diagnosed fracture, which was defined using the International Classification of Diseases, 10th revision (ICD-10) codes: vertebral fracture (S22.0, S22.1, S32.0, M48.4 and M48.5), hip fracture (S72.0 and S72.1) and other fractures—upper arm (S42.0, S42.2, and S42.3), forearm (S52.5 and S52.6), and lower leg (S82.3, S82.5, and S82.6).

The matched control participants were evaluated at the same time as each cholecystectomy group participant (index date). Patients who died or suffered fractures within 1y following cholecystectomy and patients who had a fracture before the index date were excluded.

### Definition of Comorbidity and Other Variables

Age groups were divided into three groups based on 50 y for imposing menopause and 65 y for the elderly. The income status was classified by the insurance premium and those with premiums of < 20% and Medicaid eligibility were defined as low-income group. The place of residence was categorized as metropolitan, city, or rural area. Regular exercise was defined as engaging in vigorous exercise on a regular basis (activity periods of high-intensity more than three times per week or activity periods of moderate intensity more than five times per week) (24). Body mass index (BMI) were also recorded, and participants were considered obese when the BMI was ≥ 25 kg/m^2^ based on the criteria for the Asian-Pacific region (25). Diabetes mellitus (DM) was defined based on insulin or oral hypoglycemic agent use, or a fasting plasma glucose level ≥ 126 mg/dl (26). Participants were diagnosed as hypertensive if the systolic pressure was ≥ 140 mmHg, if the diastolic pressure was ≥ 90 mmHg, or if current antihypertensive medication was used (27, 28). Dyslipidemia was defined as serum total cholesterol ≥ 240 mg/dl or use of lipid-lowering drugs (29). The characteristics of the participants undergoing cholecystectomy were subsequently analyzed and stratified according to the age group, with or without comorbidity.

### Statistical Analyses

Baseline characteristics of the study population were presented using descriptive statistics. Data are presented as mean ± standard deviation for continuous variables and as proportions for categorical variables. Continuous variables were evaluated using analysis of variance (ANOVA), and categorical variables were evaluated using the χ^2^ test or Fisher’s exact test. A Cox proportional hazards model was used to determine the independent effect of cholecystectomy on fracture risk, after adjustments for age (continuous), sex, income, place of residence, BMI, smoking status, alcohol consumption, physical activity, and comorbidities. The incidence of fracture between the two groups was calculated per 1,000 person-years.

All the statistical analyses were performed using SAS version 9.4 (SAS Institute, Cary, NC, United States) and R version 3.2.3 (The R Foundation for Statistical Computing, Vienna, Austria, http://www.Rproject.org). A two-tailed *P*-value of <0.05 was considered to indicate statistical significance.

## Results

### Characteristics of the Study Population

A total of 143,667 patients with cholecystectomy and 255,522 matched comparison participants were finally analyzed and followed up until 2016; the average follow-up period was 2.64 years. The baseline characteristics at the enrolment are described in **Table 1**.

**Table 1:** Demographics of the study population.

n	Matched controls	Cholecystectomy cohort	*P*-value^†^
	n= 255,522	n = 143,667	
**Age (mean ± SD), years**	57.92 ± 10.91	57.40 ± 10.92	<0.0001
**Age category, years**			<0.0001
40-49	63,923 (25.02)	38,223 (26.61)	
50-64	118,359 (46.32)	66,901 (46.57)	
≥65	73,240 (28.66)	38,543 (26.83)	
**Number of male (%)**	135,916 (53.19)	76,400 (53.18)	0.9371
**Low-income individuals (%)^¶^**	53,094 (20.78)	29,190 (20.32)	0.0006
**Place of residence**			<0.0001
Metropolitan	155,482 (60.85)	87,166 (60.67)	
City	70,353 (27.53)	40,469 (28.17)	
Rural	29,687 (11.62)	16,032 (11.16)	
**Comorbidity**			
**Diabetes mellitus**	36,639 (14.34)	26,532 (18.47)	<0.0001
**Hypertension**	98,155 (38.41)	60,688 (42.24)	<0.0001
**Dyslipidemia**	72,321 (28.30)	44,767 (31.16)	<0.0001
**Obesity**	87,986 (34.43)	61,869 (43.06)	<0.0001
**Current smoker (%)**	48,013 (18.79)	28,110 (19.57)	<0.0001
**Heavy drinker (%)** ^‡^	17,091 (6.69)	9,475 (6.60)	0.255
**Regular exercise (%)** ^††^	55,873 (21.87)	29,508 (20.54)	<0.0001
**Body weight**	23.97 ± 3.06	24.66 ± 3.23	<0.0001
**Cholesterol**	197.00 ± 37.50	195.30 ± 38.30	<0.0001
**DBP**	76.94 ± 9.97	77.06 ± 9.85	0.0003
**Glucose**	101.50 ± 25.03	103.53 ± 27.00	<0.0001
**SBP**	124.60 ± 15.29	124.59 ± 14.99	0.9942

^†^P-value was calculated using Student’s t-test for the continuous variables or the χ^2^ test for the categorized variables. ^‡^Heavy drinkers were those with alcohol consumption over 30g/day. ^††^Regular exercise was defined as a person who performs ≥5 moderate-intensity exercises or ≥3 vigorous-intensity exercises per week. ^¶^Low-income population was defined as the case where the income level falls in the bottom 20% of the health insurance premium. SD, standard deviation; DBP, diastolic blood pressure; SBP, systolic blood pressure.

The mean age was 57.4 years, and 53.18% of the case population comprised men. The cholecystectomy group included a higher proportion of individuals with hypertension, diabetes mellitus, or dyslipidemia than the control group. In the cholecystectomy group, 42.24% had hypertension, 18.47% had diabetes mellitus, and 31.16% had dyslipidemia (**Table 1**). When compared with the control group, a higher proportion of individuals in the cholecystectomy group was current smoker and had obesity. There was no significant difference in the ratio of heavy drinkers in both the groups.

### Risk of Fractures After Cholecystectomy

The incidence of all fractures in the cholecystectomy group was 14.689 cases per 1,000 person-year and that in the control group was 13.862 cases per 1,000 person-year (**Table 2**). The adjusted hazard ratio (aHR) for all fractures in the cholecystectomy group versus the control group was 1.095 (95% confidence interval (CI) 1.059–1.132, *p* = 0.0006). Similar tendency was observed in vertebral fractures and hip fractures (**Table 2**). The incidence of vertebral fractures and hip fractures in the cholecystectomy group were 6.483 and 1.228 cases per 1,000 person-year respectively comparing with 5.976 and 1.019 cases per 1,000 person-year, respectively, in the control group. The aHR for vertebral fractures and hip fractures in the cholecystectomy group versus the control group were 1.134 (95% CI 1.078–1.193) and 1.283 (95% CI 1.139–1.444).

**Table 2:** Incidence of fractures in cholecystectomy group and control group.

		n	Event	Person-years	Incidence rate^†^	Crude HR (95% CI)	Age/sex adjusted HR (95% CI)	All adjusted HR (95% CI)^‡^
All	Control	255522	9374	676261.39	13.8615	1 (Ref.)	1 (Ref.)	1 (Ref.)
	Cholecystectomy	143667	5526	376190.77	14.6894	**1.060 (1.026,1.096)**	**1.093 (1.057,1.130)**	**1.095 (1.059,1.132)**
Vertebra	Control	255522	4041	676261.39	5.9755	1 (Ref.)	1 (Ref.)	1 (Ref.)
	Cholecystectomy	143667	2439	376190.77	6.48341	**1.085 (1.032,1.141)**	**1.129 (1.073,1.187)**	**1.134 (1.078,1.193)**
Hip	Control	255522	689	676261.39	1.01884	1 (Ref.)	1 (Ref.)	1 (Ref.)
	Cholecystectomy	143667	462	376190.77	1.2281	**1.206 (1.072,1.356)**	**1.264 (1.124,1.422)**	**1.283 (1.139,1.444)**

^†^Fracture incidence per 1,000 person-years, ^‡^adjusted for age, sex, income, place of residence, diabetes mellitus, hypertension, dyslipidemia, smoking, alcohol drinking, exercise, and body mass index; diabetes mellitus defined as using insulin or oral hypoglycemic agents, or a fasting plasma glucose level ≥ 126 mg/dl; hypertension defined as systolic pressure was ≥ 140 mm Hg, or the diastolic pressure was ≥ 90 mmHg, or if current antihypertensive medication was used; dyslipidemia defined as serum total cholesterol was ≥ 240 mg/dl or as using lipid-lowering drugs. HR, hazard ratio; CI, confidence interval; Ref, reference. Bold style indicates statistical significance.

### Subgroup Analysis

Subgroup analyses were subsequently performed according to the age and sex (**Table 3**). In men, the association between cholecystectomy and fracture risk was significant in all age groups. In contrast, in women, history of cholecystectomy increased the risk only in the elderly (≥ 65 years old) (aHR [95% CI]: 1.092 [1.029–21.160], **Table 3** **)**. When the risk was assessed in terms of place of residence, fracture risk was greatly increased following cholecystectomy in urban populations than in rural residents (aHR [95% CI]: 1.106 [1.058–1.157], 1.121 [1.054–1.193), and 1.015 [0.932–1.105] for metropolitan, city, and rural residents, respectively, **Table 3**).

**Table 3:** Incidence of all fractures in cholecystectomy group and control group according to age and sex subgroups.

Group			n	Event	Person-years	Incidence rate^†^	Crude HR(95% CI)	Age/sex adjusted HR(95% CI)	All adjusted HR(95% CI)^‡^
All		Control	255,522	9,374	676261.39	13.8615	1 (Ref.)	1 (Ref.)	1 (Ref.)
		Cholecystectomy	143,667	5,526	376190.77	14.6894	**1.060 (1.026,1.096)**	**1.093 (1.057,1.130)**	**1.095 (1.059,1.132)**
Place	Metropolitan	Control	155,482	5,109	412959.82	12.3717	1 (Ref.)	1 (Ref.)	1 (Ref.)
		Cholecystectomy	87,166	3,080	229683.58	13.4098	**1.084 (1.036,1.133)**	**1.108 (1.060,1.159)**	**1.106 (1.058,1.157)**
	City	Control	70,353	2,674	185004.62	14.4537	1 (Ref.)	1 (Ref.)	1 (Ref.)
		Cholecystectomy	40,469	1,635	104746.83	15.6091	**1.080 (1.016,1.149)**	**1.115 (1.048,1.186)**	**1.121 (1.054,1.193)**
	Rural	Control	29,687	1,591	78296.95	20.3201	1 (Ref.)	1 (Ref.)	1 (Ref.)
		Cholecystectomy	16,032	811	41760.35	19.4203	0.956 (0.879,1.041)	1.006 (0.924,1.095)	1.015 (0.932,1.105)
Male	Age, year								
	40-49	Control	31,319	373	84378.77	4.4205	1 (Ref.)	1 (Ref.)	1 (Ref.)
		Cholecystectomy	18,903	259	50537.75	5.1249	1.159 (0.989,1.359)	1.162 (0.992,1.362)	**1.182 (1.008,1.387)**
	50-64	Control	61,140	1,235	164685.27	7.4992	1 (Ref.)	1 (Ref.)	1 (Ref.)
		Cholecystectomy	34,536	781	91514.18	8.5342	**1.138 (1.041,1.245)**	**1.145 (1.047,1.252)**	**1.152 (1.053,1.260)**
	≥65	Control	43,457	1,978	110114.02	17.9632	1 (Ref.)	1 (Ref.)	1 (Ref.)
		Cholecystectomy	22,961	1,103	56757.21	19.4337	**1.082 (1.006,1.165)**	**1.078 (1.001,1.160)**	**1.115 (1.035,1.201)**
Female	Age, year								
	40-49	Control	32,604	386	87901.37	4.3913	1 (Ref.)	1 (Ref.)	1 (Ref.)
		Cholecystectomy	19,320	270	51767.7	5.2156	**1.186 (1.015,1.386)**	**1.192 (1.020,1.392)**	1.148 (0.980,1.344)
	50-64	Control	57,219	2,435	153920.15	15.8199	1 (Ref.)	1 (Ref.)	1 (Ref.)
		Cholecystectomy	32,365	1,431	86667.87	16.5113	1.044 (0.978,1.115)	1.052 (0.986,1.123)	1.044 (0.978,1.115)
	≥65	Control	29,783	2,967	75261.81	39.4224	1 (Ref.)	1 (Ref.)	1 (Ref.)
		Cholecystectomy	15,582	1,682	38946.05	43.1879	**1.095 (1.032,1.163)**	**1.091 (1.028,1.158)**	**1.092 (1.029,1.160)**

^†^Fracture incidence per 1,000 person-years, ^‡^adjusted for age, sex, income, place of residence, diabetes mellitus, hypertension, dyslipidemia, smoking, alcohol drinking, exercise, and body mass index; diabetes mellitus defined as using insulin or oral hypoglycemic agents, or a fasting plasma glucose level ≥ 126 mg/dl; hypertension defined as systolic pressure was ≥ 140 mmHg, or the diastolic pressure was ≥ 90 mmHg, or if current antihypertensive medication was used; dyslipidemia defined as serum total cholesterol was ≥ 240 mg/dl or as using lipid-lowering drugs. HR, hazard ratio; CI, confidence interval; Ref, reference. Bold style indicates statistical significance.

Interestingly, the tendency of a higher aHR in the younger age group was more pronounced in vertebral fractures (**Table 4**); the aHR [95% CI] for vertebral fracture in the cholecystectomy group was 1.366 [1.082–1.724] in the range of 40–49 years, compared to 1.126 [1.069–1.185] in 50–64 years, 1.132 [1.063–1.206] over 65 y, and 1.134 [1.078–1.193] in all ages. In contrast, in hip fractures, there were no differences between the stratification by age group (**Table 5**).

**Table 4:** Incidence of vertebral fracture in cholecystectomy group and control group according to age and sex subgroups.

Group		n	Event	Person-years	Incidence rate^†^	Crude HR (95% CI)	Age/sex adjusted HR (95% CI)	All adjusted HR (95% CI)^‡^
All	Control	255522	4041	676261.39	5.9755	1 (Ref.)	1 (Ref.)	1 (Ref.)
	Cholecystectomy	143667	2439	376190.77	6.48341	**1.085 (1.032,1.141)**	**1.129 (1.073,1.187)**	**1.134 (1.078,1.193)**
Age, year								
40-49	Control	63923	159	172280.14	0.92292	1 (Ref.)	1 (Ref.)	1 (Ref.)
	Cholecystectomy	38223	133	102305.44	1.30003	**1.407 (1.118,1.772)**	**1.407 (1.118,1.772)**	**1.366 (1.082,1.724)**
50-64	Control	191599	3882	503981.25	7.70267	1 (Ref.)	1 (Ref.)	1 (Ref.)
	Cholecystectomy	105444	2306	273885.32	8.41958	**1.093 (1.038,1.151)**	**1.09 (1.036,1.148)**	**1.126 (1.069,1.185)**
≥65	Control	73240	2651	185375.83	14.3007	1 (Ref.)	1 (Ref.)	1 (Ref.)
	Cholecystectomy	38543	1526	95703.27	15.9451	**1.115 (1.047,1.188)**	**1.114 (1.046,1.186)**	**1.132 (1.063,1.206)**
Sex								
Male	Control	135916	1585	359178.05	4.41285	1 (Ref.)	1 (Ref.)	1 (Ref.)
	Cholecystectomy	76400	941	198809.15	4.73318	1.074 (0.990,1.164)	**1.116 (1.030,1.201)**	**1.149 (1.059,1.246)**
Female	Control	119606	2456	317083.33	7.7456	1 (Ref.)	1 (Ref.)	1 (Ref.)
	Cholecystectomy	67267	1498	177381.62	8.44507	**1.090 (1.022,1.162)**	**1.137 (1.066,1.212)**	**1.128 (1.058,1.204)**

^†^Fracture incidence per 1,000 person-years, ^‡^adjusted for age, sex, income, place of residence, diabetes mellitus, hypertension, dyslipidemia, smoking, alcohol drinking, exercise, and body mass index; diabetes mellitus defined as using insulin or oral hypoglycemic agents, or a fasting plasma glucose level ≥ 126 mg/dl; hypertension defined as systolic pressure was ≥ 140 mm Hg, or the diastolic pressure was ≥ 90 mmHg, or if current antihypertensive medication was used; dyslipidemia defined as serum total cholesterol was ≥ 240 mg/dl or as using lipid-lowering drugs. HR, hazard ratio; CI, confidence interval; Ref, reference. Bold style indicates statistical significance.

**Table 5:** Incidence of hip fracture in cholecystectomy group and control group according to age and sex subgroups.

Group		n	Event	Person-years	Incidence rate^†^	Crude HR (95% CI)	Age/sex adjusted HR (95% CI)	All adjusted HR (95% CI)^‡^
All	Control	255522	689	676261.39	1.01884	1 (Ref.)	1 (Ref.)	1 (Ref.)
	Cholecystectomy	143667	462	376190.77	1.2281	**1.206 (1.072,1.356)**	**1.264 (1.124,1.422)**	**1.283 (1.139,1.444)**
Age, year								
40-49	Control	63923	23	172280.14	0.1335	1 (Ref.)	1 (Ref.)	1 (Ref.)
	Cholecystectomy	38223	16	102305.44	0.15639	1.171 (0.619,2.217)	1.168 (0.617,2.211)	1.290 (0.679,2.454)
50-64	Control	191599	666	503981.25	1.32148	1 (Ref.)	1 (Ref.)	1 (Ref.)
	Cholecystectomy	105444	446	273885.32	1.62842	**1.233 (1.093,1.39)**	**1.232 (1.093,1.389)**	**1.282 (1.136,1.446)**
≥65	Control	73240	547	185375.83	2.95076	1 (Ref.)	1 (Ref.)	1 (Ref.)
	Cholecystectomy	38543	349	95703.27	3.64669	**1.237 (1.081,1.415)**	**1.236 (1.081,1.414)**	**1.232 (1.076,1.411)**
Sex								
Male	Control	135916	352	359178.05	0.98002	1 (Ref.)	1 (Ref.)	1 (Ref.)
	Cholecystectomy	76400	239	198809.15	1.20216	**1.227 (1.041,1.446)**	**1.286 (1.091,1.515)**	**1.360 (1.152,1.605)**
Female	Control	119606	337	317083.33	1.06281	1 (Ref.)	1 (Ref.)	1 (Ref.)
	Cholecystectomy	67267	223	177381.62	1.25718	1.182 (0.998,1.004)	**1.237 (1.044,1.465)**	**1.218 (1.028,1.444)**

^†^Fracture incidence per 1,000 person-years, ^‡^adjusted for age, sex, income, place of residence, diabetes mellitus, hypertension, dyslipidemia, smoking, alcohol drinking, exercise, and body mass index; diabetes mellitus defined as using insulin or oral hypoglycemic agents, or a fasting plasma glucose level ≥ 126 mg/dl; hypertension defined as systolic pressure was ≥ 140 mmHg, or the diastolic pressure was ≥ 90 mm Hg, or if current antihypertensive medication was used; dyslipidemia defined as serum total cholesterol was ≥ 240 mg/dl or as using lipid-lowering drugs. HR, hazard ratio; CI, confidence interval; Ref, reference. Bold style indicates statistical significance.

In contrast, there was no difference in the risk of fractures according to comorbidity such as diabetes, hypertension, hyperlipidemia, BMI, regular exercise (**Supplementary Table S1**).

## Discussion

In the present study, we have demonstrated that the incidence of all fractures was higher in the participants who underwent a cholecystectomy than their age-sex-matched controls (aHR = 1.095). The incidence rate of all fractures was 14.689 cases per 1000 person-years following cholecystectomy, whereas it was 13.861 cases per 1000 person-years in controls *(p* = 0.0006). The incidence of vertebral fractures in the cholecystectomy group and control group was 6.483 and 5.976 cases per 1,000 person-year (aHR = 1.134). In hip fractures, the incidence rate were 1.228 and 1.019 cases per 1,000 person-year in each group (aHR = 1.283).

The strength of this tendency was different considering age and sex. In all fractures, the risk was not different regardless of age in men, while it was significant in the elderly (≥65 y) in women (**Table 3**). In addition, we found that the fracture risk was more affected after cholecystectomy in urban populations than in rural residents. In view of vitamin D synthesis, this could be related to rural residents doing more outdoor activities (sun exposure) than those in urban populations. Among fracture sites, cholecystectomy increased the risk of vertebral fracture in the younger age group; however, there was no interaction between cholecystectomy and age in hip fractures (**Tables 4** and **5**).

To the best of our knowledge, this is the first study to investigate the incidence of fracture in participants who underwent cholecystectomy. To date, only a few published papers have evaluated the possible relationship between osteoporosis and cholecystectomy (20–23, 30). Osteoporosis is a common health problem with an increased financial burden; osteoporotic fracture is the most worrying complication of osteoporosis. In previous studies, serum vitamin D levels following cholecystectomy were studied as a possible factor increasing the risk of osteoporosis (20–22). Adequate calcium and vitamin D intake play a key role in not only optimal peak bone mass development but also bone mass preservation throughout life (13). Only 10-15% of dietary calcium and approximately 60% of phosphorus is absorbed without vitamin D (13). Vitamin D deficiency can accelerate and exacerbate osteopenia, osteoporosis, and fractures in adults (31–33). In Korea, vitamin D status is still deteriorating, and vitamin D deficiency is a fairly common health problem (34, 35). In the susceptible population, cholecystectomy may play an important role in the risk of fracture.

There is no known exact mechanism regarding the fracture risk following cholecystectomy, but vitamin D deficiency is more prevalent when gastrointestinal diseases are present or after surgery associated with them (21, 36, 37). Vitamin D is lipophilic, so bile acids are required for proper intestinal absorption, which may be impaired after cholecystectomy; the bile released from the liver flows continuously and irrepressibly into the duodenum (38, 39). Considering this, there might be changes in the vitamin D synthesis or metabolism in patients who underwent cholecystectomy; thus, this could be one of the possible explanations for increased fracture risk following cholecystectomy.

On the other hand, changes in bile flow following cholecystectomy can affect the gut microbiota and its diversity (40–42). Gut microbiota comprises the largest number of cells (10^14^) within the intestinal tract lumen and its composition and/or products affect the bone homeostasis (43). In germ-free mice that do not have microbiota, bone mass is increased compared to conventionally raised mice. Colonization of germ-free mice with a normal gut microbiota normalizes bone mass by normalizing the frequency of CD4þ T cells and CD11bþ/GR 1 osteoclast precursor cells in the bone marrow (44). The association of gut microbiota with bone metabolism is further supported by studies demonstrating that antibiotic, probiotic, and prebiotic treatments impact gut microbiota composition and regulate bone metabolism (45). Similar results were demonstrated in human studies (46–49). Considering this, alteration in gut microbiota caused by cholecystectomy could be associated with the increased fracture risk through bone mass regulation by immune system mediation, which in turn regulated osteoclastogenesis, thereby disturbing osteoblast’ and osteoclast’ activities.

Interestingly, vertebral fracture showed higher incidence in young people compared to patients aged > 65. Higher incidence in young age group could possibly be attributed to many other causes (injuries, traumas, etc.) rather than osteoporotic fractures. In contrast, the absence of any significant difference in the incidence of hip fractures between the three age groups implies that cholecystectomy could lead to trabecular framework (vertebrae) deterioration rather than cortical framework (hip) deterioration.

This has certain limitations. First, we used claim data for identifying the patients who underwent cholecystectomy. Using the code reported on the insurance claims made it difficult to distinguish a single cholecystectomy from additional excision of gall bladder during other surgeries. Moreover, the cause of cholecystectomy could not be identified. Second, ICD codes were used for defining new fractures, and thus the cause of fracture, such as traumatic, osteoporotic, or other causes, could not be defined. However, the subgroups were divided into vertebral fractures and hip fractures, which are common site of osteoporotic fractures. Third, fractures are often associated with osteoporosis, so secondary osteoporosis such as associated with the use of glucocorticoids should be considered. However, patient medication history data collection (osteoporosis, osteopenia, steroid usage, etc.) within the health insurance claim data was limited. In addition to the medical history, variables such as serum vitamin D, calcium, and parathyroid hormone levels and BMD may influence the association between cholecystectomy and fracture risk; however, they were not available in the claim database. Fourth, the average follow-up period was 2.64 y. It might be inadequate for assessment of the development of fracture among groups. Therefore, long-term follow-up studies are warranted to confirm our findings. Fifth, possible selection bias could exist since the study populations were recruited from health check-up examination. Last, the aHR for incidence of all fractures was 1.095 which means an increasing risk of 9.5%. The effect size is small but meaningful. Kim et al. (50) developed a Korean osteoporotic fractures prediction model using clinical risk factors; with the exception of age, recent fragility fracture (aHR: 3.53 in men and 1.83 in women), recent use of glucocorticoids (aHR: 1.87 in men and 1.51 in women), and current smoking (aHR: 1.08 in men and 1.15 in women) were associated with a 7-year increased risk of osteoporotic fracture. In this study, the impact of cholecystectomy on the fracture risk was similar to that of smoking. However, the susceptibility of vertebral fracture after cholecystectomy was different based on the age; the risk was significantly increased by 36.6% in younger individuals aged 40-49 y, but the risk was not that high among individuals aged ≥ 50 y (**Table 4** **)**. Therefore, our findings indicate a high-risk population for vertebral fracture following cholecystectomy. High-risk individuals may be recommended their regular vitamin D level check-up following cholecystectomy; if found deficient, they should consider vitamin D and calcium supplements administration.

The strengths of this study are apparent; we used the nationwide database to perform a population-based cohort study, compared to the small number of participants of previous studies. Moreover, this is the first study that reported a positive relationship between cholecystectomy and fractures.

To conclude, prior cholecystectomy is associated with increased risk of fractures. The risk of developing fracture is relatively high in a younger age group, especially in vertebral fractures in an age group of 40 to 49 years. Therefore, cholecystectomy should be considered only after rigorous selection of indicated patients. Further studies are necessary to elucidate the exact mechanism and potential confounding factors, such as certain medications, bone turnover markers, dietary intake of calcium and vitamin D, sunlight exposure, or trauma histories, which could have an effect on the development of fractures.

## Data Availability Statement

The data analyzed in this study is subject to the following licenses/restrictions: We used data from the Korean National Health Insurance Corporation (NHIC) database that only authorized people could access. Requests to access these data sets should be directed to KH, hkd917@naver.com.

## Ethics Statement

The studies involving human participants were reviewed and approved by IBR number: X-2003/601-904. Written informed consent for participation was not required for this study in accordance with the national legislation and the institutional requirements.

## Author Contributions

CMS, DHL, YSP: study conception; CMS, KH, NK: study design; KH, SHP: data analysis; CMS, EJL, YJK, HY: interpretation of data; EJL: manuscript drafting; CMS, DHL, KH, SHP, YJK, HY, YSP, NK: critical comments of manuscript; CMS, DHL, KH, SHP: revision of the manuscript and tables. All authors contributed to the article and approved the submitted version. All authors agreed to be accountable for all aspects of the work in ensuring that questions related to the accuracy or integrity of any part of the work are appropriately investigated and resolved.

## Funding

This research was supported by a grant of the Korea Health Technology R&D Project through the Korea Health Industry Development Institute (KHIDI), funded by the Ministry of Health & Welfare, Republic of Korea (grant number: HI18C1140).

## Conflict of Interest

The authors declare that the research was conducted in the absence of any commercial or financial relationships that could be construed as a potential conflict of interest.
