# Supplementary material for: The Association Between Cholecystectomy and the Risk for Fracture: A Nationwide Population-Based Cohort Study in Korea
Source: Front Endocrinol (Lausanne). 2021 May 27;12:657488. doi: 10.3389/fendo.2021.657488 (PMC8190474; doi:10.3389/fendo.2021.657488)
Supplement: Supplementary file 1 [file DataSheet_1.pdf]

**Supplementary Table S1.** Risk of all fractures in cholecystectomy group according to the presence or absence of comorbidities.

| | | n | Event | Person-years | Incidence rate[†] | All adjusted HR (95% CI)[‡] | Interaction P |
|---|---|---|---|---|---|---|---|
| Diabetes mellitus | | | | | | | 0.6323 |
| No | Control | 218883 | 7698 | 583102.4 | 13.2018 | 1(Ref.) | |
| | Cholecystectomy | 117135 | 4218 | 309794.81 | 13.6155 | **1.090(1.050,1.132)** | |
| Yes | Control | 36639 | 1676 | 93158.99 | 17.9907 | 1(Ref.) | |
| | Cholecystectomy | 26532 | 1308 | 66395.96 | 19.7 | **1.113(1.035,1.197)** | |
| Hypertension | | | | | | | 0.4859 |
| No | Control | 157367 | 4822 | 418220.92 | 11.5298 | 1(Ref.) | |
| | Cholecystectomy | 82979 | 2584 | 219612.56 | 11.7662 | **1.081(1.030,1.134)** | |
| Yes | Control | 98155 | 4552 | 258040.47 | 17.6406 | 1(Ref.) | |
| | Cholecystectomy | 60688 | 2942 | 156578.21 | 18.7893 | **1.109(1.059,1.162)** | |
| Dyslipidemia | | | | | | | 0.6730 |
| No | Control | 183201 | 6339 | 493707.4 | 12.8396 | 1(Ref.) | |
| | Cholecystectomy | 98900 | 3580 | 264560.24 | 13.5319 | **1.103(1.058,1.149)** | |
| Yes | Control | 72321 | 3035 | 182553.99 | 16.6252 | 1(Ref.) | |
| | Cholecystectomy | 44767 | 1946 | 111630.53 | 17.4325 | **1.080(1.020,1.143)** | |
| BMI | | | | | | | 0.7594 |
| <25 | Control | 167536 | 6194 | 442741.74 | 13.9901 | 1(Ref.) | |
| | Cholecystectomy | 81798 | 3210 | 213831.48 | 15.0118 | **1.085(1.040,1.132)** | |
| ≥25 | Control | 87986 | 3180 | 233519.65 | 13.6177 | 1(Ref.) | |
| | Cholecystectomy | 61869 | 2316 | 162359.28 | 14.2647 | **1.091(1.034,1.151)** | |
| Regular exercise | | | | | | | 0.1266 |
| No | Control | 199649 | 7616 | 529481.67 | 14.3839 | 1(Ref.) | |
| | Cholecystectomy | 114159 | 4480 | 299287.63 | 14.9689 | **1.081(1.042,1.122)** | |
| Yes | Control | 55873 | 1758 | 146779.72 | 11.9771 | 1(Ref.) | |
| | Cholecystectomy | 29508 | 1046 | 76903.14 | 13.6015 | **1.156(1.071,1.249)** | |

[†]Fracture incidence per 1,000 person-years, [‡]adjusted for age, sex, income, place of residence, diabetes mellitus, hypertension, dyslipidemia, smoking, alcohol drinking, exercise, and BMI; diabetes mellitus defined as using insulin or oral hypoglycemic agents, or a fasting plasma glucose level ≥ 126 mg/dL; hypertension defined as systolic pressure was ≥ 140 mmHg, or the diastolic pressure was ≥ 90 mmHg, or if current antihypertensive medication was used; dyslipidemia defined as serum total cholesterol was ≥ 240mg/dL or as using lipid-lowering drugs. HR, hazard ratio; CI, confidence interval; Ref, reference; BMI, body mass index. Bold style indicated statistical significance.
